# *Cyperus* spp.: A Review on Phytochemical Composition, Biological Activity, and Health-Promoting Effects

**DOI:** 10.1155/2021/4014867

**Published:** 2021-09-07

**Authors:** Yasaman Taheri, Jesús Herrera-Bravo, Luis Huala, Luis A. Salazar, Javad Sharifi-Rad, Muhammad Akram, Khuram Shahzad, Guiomar Melgar-Lalanne, Navid Baghalpour, Katayoun Tamimi, Javad Mahroo-Bakhtiyari, Dorota Kregiel, Abhijit Dey, Manoj Kumar, Hafiz Ansar Rasul Suleria, Natália Cruz-Martins, William C. Cho

**Affiliations:** ^1^Phytochemistry Research Center, Shahid Beheshti University of Medical Sciences, Tehran, Iran; ^2^Departamento de Ciencias Básicas, Facultad de Ciencias, Universidad Santo Tomas, Chile; ^3^Center of Molecular Biology and Pharmacogenetics, Scientific and Technological Bioresource Nucleus, Universidad de La Frontera, Temuco 4811230, Chile; ^4^Department of Eastern Medicine, Government College University Faisalabad, Pakistan; ^5^Instituto de Ciencias Básicas, Universidad Veracruzana, Av. Dr. Luis Castelazo Ayala s/n. Col Industrial Ánimas, 91192 Xalapa, Veracruz, Mexico; ^6^Department of Environmental Biotechnology, Lodz University of Technology, Wolczanska 171/173, 90-924 Lodz, Poland; ^7^Department of Life Sciences, Presidency University, 86/1 College Street, Kolkata 700073, India; ^8^Chemical and Biochemical Processing Division, ICAR–Central Institute for Research on Cotton Technology, Mumbai 400019, India; ^9^Department of Agriculture and Food Systems, The University of Melbourne, Melbourne 3010, Australia; ^10^Department of Biomedicine, Faculty of Medicine, University of Porto, Alameda Prof. Hernâni Monteiro, Porto, Portugal; ^11^Institute for Research and Innovation in Health (i3S), University of Porto, Porto, Portugal; ^12^Institute of Research and Advanced Training in Health Sciences and Technologies (CESPU), Rua Central de Gandra, 1317, 4585-116, Gandra, PRD, Portugal; ^13^Department of Clinical Oncology, Queen Elizabeth Hospital, Kowloon, Hong Kong

## Abstract

Cyperaceae are a plant family of grass-like monocots, comprising 5600 species with a cosmopolitan distribution in temperate and tropical regions. Phytochemically, *Cyperus* is one of the most promising health supplementing genera of the Cyperaceae family, housing ≈950 species, with *Cyperus rotundus* L. being the most reported species in pharmacological studies. The traditional uses of *Cyperus* spp. have been reported against various diseases, *viz.*, gastrointestinal and respiratory affections, blood disorders, menstrual irregularities, and inflammatory diseases. *Cyperus* spp. are known to contain a plethora of bioactive compounds such as *α*-cyperone, *α*-corymbolol, *α*-pinene, caryophyllene oxide, cyperotundone, germacrene D, mustakone, and zierone, which impart pharmacological properties to its extract. Therefore, *Cyperus* sp. extracts were preclinically studied and reported to possess antioxidant, anti-inflammatory, antimicrobial, anticancer, neuroprotective, antidepressive, antiarthritic, antiobesity, vasodilator, spasmolytic, bronchodilator, and estrogenic biofunctionalities. Nonetheless, conclusive evidence is still sparse regarding its clinical applications on human diseases. Further studies focused on toxicity data and risk assessment are needed to elucidate its safe and effective application. Moreover, detailed structure-activity studies also need time to explore the candidature of *Cyperus*-derived phytochemicals as upcoming drugs in pharmaceuticals.

## 1. Introduction

Immemorially, human societies have been using herbs and their products as sources of medicine, nutrition, and industrial applications [1]. As an example of the role of plant species in human life, in ancient Egypt, the first paper was made from papyrus (*Cyperus papyrus* L.), a species of the Cyperaceae family [2]. Cyperaceae includes grass-like monocots, comprising around 5600 species and 100 genera, and the family is widespread on all continents with the exception of Antarctica. The second largest genus in this family is *Cyperus*, with ~950 species [3]. *Cyperus* spp. are most commonly known as weeds, despite some cultures using them for medicinal purposes and as a source of food [4]. *Cyperus* spp. predominantly exist in the wetlands throughout the globe in the tropical regions and act as source of primary productivity. The tubers, shoots, and fruits of this species are produced in larger quantities and act as a source of food for amphibians and aquatic animals [5].

The traditional use of *Cyperus* plants has been reported from all over the world as a remedy against various human ailments [6] including treatment of stomach and bowel disorders, as diuretic, digestant, and lactodepurant purposes. The plant extracts also act as a selective drug for the treatment of bronchitis, blood disorders, menstrual irregularities, amenorrhea, diarrhea, dysentery, and inflammatory diseases [7]. Interestingly and despite *Cyperus* including more than 950 species, the three most commonly reported species are purple nutsedge (*Cyperus rotundus* L.), yellow nutsedge (*Cyperus esculentus* L.), and *C. papyrus*. *Cyperus rotundus* is the most well-known species of *Cyperus* in South Asia, a perennial weed that grows best in high-moisture soil and reproduces easily through rhizomes and tubers [7]. This species is indigenous to the tropical and subtropical parts of the Old World, and despite the fact that it can be found detrimental in cultivated fields, it has several beneficial uses as medicine since ancient times [8]. *Cyperus rotundus* rhizomes and tubers are mentioned in Oriental traditional medicine to treat fever, digestive disorders, and menstrual irregularities in several countries including China, India, Iran, and Japan [9, 10].

*Cyperus esculentus* L. is an edible perennial grass-like plant native to the Old World. This species exists widely throughout tropics and subtropics of North America [11]. The earliest records of its use dated back to predynastic times about 6000 y ago in North America and Egypt; however, its different varieties are mostly found in Southern Europe, South-Middle East, and Africa [12]. It has been also considered as a foodstuff since ancient times, especially in ancient Egypt. It is a crop of early domestication and was regarded important with the other crops of the Nile Valley. Its dry tubers have been found in tombs from predynastic times about 6000 y ago. *Cyperus esculentus* tubers were roasted and used as a sweetmeat in Egypt during the ancient times [12]. *Cyperus esculentus* is widely cultivated for its edible tubers, called earth almonds or tigernuts [13], which are consumed as a popular snack in Africa and for making a sweet milk-like beverage, horchata de chufa, commonly consumed in Spain and other European and Latin-American countries [14]. Tigernut is a rich source of protein and minerals making the beverage highly nutritious (phosphorus and potassium) [12].

*Cyperus papyrus* L. is an aquatic sedge mostly known for its use in the preparation of the paper by the traditional Egypt, Greek, and Roman civilizations. Paper made from dried, pressed, and woven strips of culm pith had been used since 3500 BC by ancient civilizations in the Egypt and the Mediterranean Basin. It was the only widespread recording medium until the 8^th^ century in Europe [15]. Other species of the *Cyperus* family include *Cyperus compressus* L., *Cyperus javanicus* Houtt., and *Cyperus monocephalus* Roxb. (*Cyperus cephalotes*). For instance, *C. compressus* is a grass-like plant and is widely distributed across the tropical and subtropical regions of the world. In India, the powdered roots of *C. compressus* have long been used in traditional medicine by the Santhal tribes to treat intestinal helminthic infections [16]. Examples of folk medicinal and edible uses of *Cyperus* spp. reported from different parts of the world are briefly shown in Table 1.

**Figure 1:**
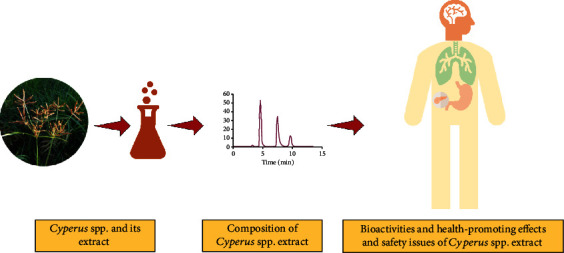
Diagram showing various components discussed in the review.

Taken together, the multiple potentialities reported so far for the most widely exploited *Cyperus* spp. were considered. This review is the first of its kind that gives a comprehensive discussion on the recent findings related to chemical composition, biological activities, and pharmacological effects of such promissory naturally occurring matrices. The safety and toxicity effects of the *Cyperus* sp. extracts are also considered in the scope of the manuscript. The diagram showing various components discussed in the review are presented in Figure 1.

## 2. Chemical Composition

The Cyperaceae family is one of the largest flowering plant families and is ranked the third largest monocot family after Orchidaceae and Poaceae [46]. A rising number of studies have highlighted that the multiple potentialities of the species of this family as medicines are attributed to the presence of several bioactive constituents. For example, the cypriol, isolated from *Cyperus scariosus* R.Br. essential oil, is present in various perfumes and medicines. In fact, cypriol's ambery, balsamic, spicy, warm, and woody features make it highly demanded in perfume industry [47]. In addition, the essential oil is also present in various other species of *Cyperus*, such as *C. articulatus* L., *C. rotundus*, and *Cyperus maculatus* Boeckeler [48]. Summarization of phytochemicals present in the major six species of the Cyperus genus is summarized in Table 2, and in the next subsections, a brief description of the most abundant phytochemicals in the recently investigated *Cyperus* spp. is also presented.

### 2.1. *Cyperus articulatus* L.

*Cyperus articulatus* is a perennial herb with underground perennial rhizomes having scales which grade into culm leaves. They have exceptionally high photosynthesizing function compared to other plants and are also regarded as herbal switch plants as they are a reservoir of potentially useful drugs for the treatment metabolic disorders [49]. Various specific compounds isolated from *C. articulatus* include *α*-cyperone, *α*-corymbolol, *α*-pinene, caryophyllene oxide, cyperotundone, and mustakone. Researchers also identified articulone, myrtenal, and myrtenol from volatile oil of Nigerian *C. articulatus* [55]. Cameroonian *C. articulatus* hexane extracts displayed the presence of isopatchoul-4(5)en-3-one, mandassidione, mustakone, and almost all sesquiterpene diketones [56]. Similar compounds were also identified in the Brazilian rhizome volatile oil with mustakone (14%), caryophyllene oxide (10.2%), and *α*-pinene (6.4%) [57]. Volatile oil from *C. articulatus* rhizomes showed the presence of *α*-pinene (3.5–25.2%), *β*-pinene (2.3–12.6%), *trans*-pinocarolol (2.2–5.5%), myrtenal + myrtenol (2.3–5.6%), *α*-copaene (1.3–2.6%), cyperene (0.7–1.6%), *β*-selinene (0.8–2.4%), lithol (0.9–5.1%), caryophyllene oxide (3.1–8.3%), mustakone (3.4–9.9%), cyperotundone (2.6–4.1%), and *α*-cyperone (3.2–8.8%) [58]. These reports suggest a qualitative and quantitative difference in the volatile oil composition. The difference was attributed to various factors like air pollution, altitude, harvesting time, developmental stage, luminosity, seasonality, temperature, water availability, nutrients, UV radiation, and pathogens [59, 60].

### 2.2. *Cyperus conglomeratus* Rottb.

*Cyperus conglomeratus* is a perennial monocot with coarse rhizomes up to 12−16 cm long and 0.2–0.3 cm in width. It is native to India but also grows in temperate, tropical, and subtropical regions [61]. It is a perennial weedy herb commonly found with slim and sheathing leaf base. Phytochemical analysis of several plant extracts revealed the existence of different types of constituents at different amounts with medicinal activities. For example, the crude powder is rich in steroids, while the solvent extract is rich in triterpenes [62]. The therapeutic efficacy was not limited to a specific plant part; every plant part displays a pharmacological activity. Henceforth, the pharmacognostic studies of different plant parts have been performed for different plant organs [63], such as aerial parts, flower, fruit, leaf root, and stem [64–71]. The fatty acid profile of *C. conglomeratus* showed the presence of palmitic, linoleic, heptadecanoic, oleic, myristic, arachidonic, lignoceric, and stearic acid. The unsaponifiable fractions of *C. conglomeratus* constituted two other bioactive compounds (*β*-sitosterol and *α*-amyrin).

### 2.3. *Cyperus distans* L.f.

*Cyperus distans* is an annual herb of about 0.6–1.5 m tall, mostly found in humid areas along roadsides and rivers and as weeds [72]. The phytochemical study of *C. distans* revealed the presence of scabequinone with antifeeding effects [73]. From 80% of its oil composition, almost 22 compounds were isolated, being zierone (33.8%) the main component. Other noteworthy compounds were caryophyllene oxide (14.1%), *α*-cyperone (9.1%), humulene epoxide II (3.8%), cyperene (3.2%), endesma-2,4,11-triene (2.9%), nor-copernone (2.9%), and germacrene D (2.8%) [74–77].

### 2.4. *Cyperus esculentus* L.

*Cyperus esculentus*, also known as yellow nutsedge, is a perennial herb growing in tropical and temperate regions of the world. Naturally, it is found as a weed in farming areas and in wastelands [78]. The taste of tubers is sweet and reported to have health and nutritional benefits [79]. The common names of *C. esculentus* are chufas, earth almond, nutsedge, and rush nut [80]. Cyprotundone, the volatile component p-vinylguaiacol (2-methoxy-4-vinylphenol) [81], and vanillin (4-hydroxy-3-methoxy benzaldehyde) have been identified [82], with interesting bioactive effects that been reported to these biomolecules. As a matter of fact, these molecules are helpful in tracing components in cosmetics, dairy products, drug preparations, and pastry products [83].

### 2.5. *Cyperus longus* L.

*Cyperus longus* is an Egyptian plant, distributed throughout Africa and Europe and to Indian subcontinent and is used as herbal tonic and diuretic [84]. Compounds isolated from this plant are flavonoids, stilbenes, and terpenoids [85–88]. Other compounds from *C. longus* essential oil were also identified such as *β*-himachalene (46.6%), *α*-humulene (16.7%), and *γ*-himachalene (10.1) as main components [86]. In another study, 32 components were identified consisting 83.50% of essential oil using gas chromatography-mass spectroscopy (GC-MS) analysis [53].

### 2.6. *Cyperus rotundus* L.

**Figure 2:**
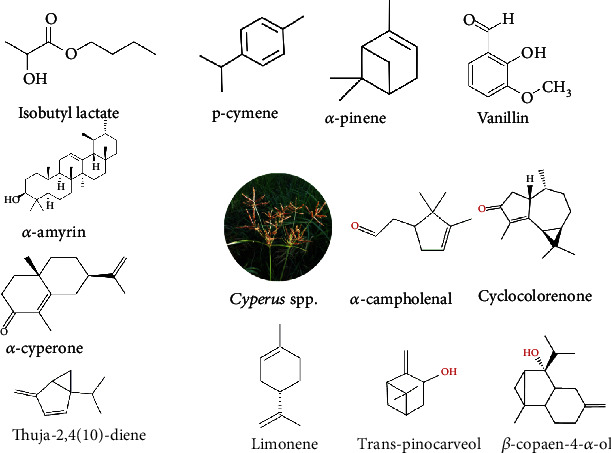
Structure of important members of bioactive compounds from *Cyperus* spp.

*Cyperus rotundus* is popularly known as Nagarmotha or purple nutsedge or nut grass [89]. This is a perennial herb with creeping rhizomes 1–3 cm long having a bulbous base. The stems of this herb can attain as the size of about 140 cm, and leaves are grooved on the upper surface. The ethanolic extracts of *C. rotundus* were determined using HPLC, and it was reported to contain two bioactive phenolics, i.e., quercetin and chlorogenic acid [90]. Structures of important members of bioactive compounds from *Cyperus* spp. are shown in Figure 2.

## 3. Bioactive Effects: Preclinical Evidence

### 3.1. Antioxidant Activity

Antioxidants are substances which remove reactive species or free radicals from cells and play a crucial role in maintaining the health and by preventing the diseases. The antioxidant capacity of *Cyperus* spp. is attributed to the plethora of phytochemicals present. Phenolic compounds, specifically flavonoids, tannins, and coumarins, are present in this species. The presence of these phytochemicals is directly correlated with antioxidant effects [91]. For example, a study assessed the nutritional value, mineral composition, secondary metabolites, and antioxidant activity of 5 wild geophytes: 2 from the Cyperaceae family (*Cyperus capitatus* Vand. and *C. conglomeratus* Rottb.) and 3 from the Poaceae family (*Elymus farctus* (Viv.) Runemark ex Melderis, *Lasiurus scindicus* Henrard, and *Panicum turgidum* Forssk.) collected from the Egyptian coastal desert (Mediterranean coast of the Delta) and the interior desert (Wadi Hagoul). Strong radical scavenging activity with EC_50_ < 1 mg/ml assessed using the 2,2-diphenyl-1-picrylhydrazyl (DPPH) assay was reported from the extracts of *C*. *conglomeratus* and *C. capitatus* [92]. An experiment assessing the antioxidant activity of the extracts of *Cyperus tegetum* Roxb. demonstrated significant DPPH radical, superoxide anion, and hydrogen peroxide scavenging activities compared to the standards, viz., hydroxybutylanisol, butylhydroxytoluene, and ascorbic acid, respectively [93]. In addition, the milk extracted from *C. esculentus* tubers, commonly known as tigernut, was utilized in a study on rats to assess its effect on preventing acetaminophen-induced liver damage (APAP). Its presence led to an increased activity of antioxidant enzyme superoxide dismutase (SOD), while malondialdehyde concentrations were lower than the control group, thus demonstrating a good antioxidant activity [94]. The improvement in the levels of antioxidant enzymes resulted in the decreased reactive species and free radical in acetaminophen-treated liver, thus, managing the situation of liver damage. Similarly, the polyphenolic content of aquatic extracts from *C. rotundus* tubers was found to have protective effects on liver and kidney function caused by exposure to heavy metals (cadmium chloride) in rats, through scavenging of free radicals [95]. Also, components of the essential oils of *C. articulatus* rhizome encapsulated in chitosan nanoparticles revealed a high potential to eliminate free radicals [96]. The encapsulation improves the stability and also the efficiency of extracts from Cyperus spp. resulting in decreasing the stress imposed by free radicals.

*Cyperus* plant extracts have proven to have a neuroprotective effect caused due to reactive oxygen species (ROS). The deposition of beta-amyloid in the hippocampus promotes oxidative stress, reactive ROS formation, reduction of the antioxidant enzymes activity, and consequently, neuronal death. Previous studies have shown that flavonoids can modulate the function of immune cells, exerting a direct effect against inflammation and oxidative stress [97]. Thus, the antioxidant activity showed by the flavonoids present in *C. rotundus* extracts explains the increase in hippocampal neurogenesis of beta-amyloid in rat models and consequently improves the memory [98]. Orientin, a flavonoid found in *C. esculentus*, decreased oxidative stress generating a neuroprotective effect against cerebral ischemia/reperfusion injury in Sprague-Dawley rats through the middle cerebral artery occlusion method [99].

### 3.2. Anti-Inflammatory Activity

Numerous studies have proven the potent anti-inflammatory activity of extracts obtained from various plant parts of the *Cyperus* genus [100]. The anti-inflammatory action of the extract from *C. rotundus* rhizome was first described in 1971 [101], and since then, investigations have been done to confirm and understand the anti-inflammatory effect of the different plant parts or active constituents of *C. rotundus*. The compound *α*-cyperone, one of the main phytochemicals found in *C. rotundus* oil, was found to inhibit lipopolysaccharide- (LPS-) stimulated inflammatory response in a murine BV-2 microglial cell line, by activating Akt (protein kinase B)/nuclear factor-E2-related factor (Nrf)-2/heme oxygenase- (HO-) 1 and suppressing the nuclear factor kappa light chain enhancer of the activated B cell (NF-*κ*B) pathway [102]. A study concluded that *α*-cyperone exerts a neuroprotective activity by attenuating the production of inflammatory cytokines in BV-2 cells through activating Akt/Nrf2/HO-1 and suppressing the NF-*κ*B pathway.

Another study using methanol extracts from *C. rotundus* rhizomes revealed that cyperalin A has high anti-inflammatory activity through inhibition of prostaglandin E2 (PGE-2), cyclooxygenase-2 (COX-2), and arachidonate 5-lipoxygenase (LOX-5) and that sugetriol triacetate, another compound of biological interest in *C. rotundus*, presented a similar effect on PGE-2, COX-2, and LOX-5 enzymes in peripheral blood mononuclear cell (PBMC) lines [103]. *α*-Cyperone revealed to suppress the inflammatory response in lipopolysaccharide- (LPS-) induced acute lung injury in mice, through inhibiting the growth of inflammatory cells along with cytokines and downregulating the NF-*κ*B and NLR family pyrin domain containing 3 (NLRP3) signalling pathways [104]. Moreover, recent evidence has shown that the topical application of C*. rotundus* rhizome extract in a rat model with chronic and acute dermatitis leads to a reduction in ear oedema and inflammatory cell infiltration generated by exposure to 12-*O*-tetradecanoylforbol-acetate (TPA). This ultimately suggested that the extract could be a potential new therapeutic tool for the treatment of inflammatory skin disorders [90].

### 3.3. Antimicrobial Activity

The antimicrobial activity of *C. rotundus* extract has been shown in numerous studies [7, 100, 105–109]. In general, it was documented that Gram-positive bacteria were more sensitive to *Cyperus* extracts than Gram-negative bacteria. However, direct comparison of different studies was difficult due to variety of microbiological tests, microbial genera and species, presence of saccharides, herb cultivation conditions, extraction methods, and so on [110]. For example, a study carried out with *C. articulatus* essential oils revealed interesting inhibitory effects on *Staphylococcus aureus* and *Escherichia coli* [96]. Traditional medicine practitioners make use of water primarily as a solvent, but studies have shown that alcohol extracts of plants are much potent and efficacious [111]. The effects of aqueous and alcohol extracts with essential oils from *C. rotundus* tubers on cultures of *Streptococcus mutans*, *Aggregatibacter actinomycetemcomitans*, and *Candida albicans* were investigated. Alcoholic extracts displayed marked inhibition of *S. mutans* and *A. actinomycetemcomitans* growth, making these extracts future candidates for both treatment and prevention of periodontitis and oral cavity affections [105].

Another study used chloroform extracts of *C. conglomerates*, orally administered to mice, aiming to determine the degree of consumption toxicity. There was no damage to the liver or kidney at the doses used, and a negative impact on the growth of *C. albicans*, *C. dubliniensis*, *C. famata*, *C. glabrata* and *C. inconspicua* was listed. *β*-Sitosterol and *α*-amyrin were the most abundant components identified and were the chemicals responsible for the significant effect on the growth of *C. famata* and *C. albicans*, respectively [106]. *C. conglomeratus* chloroform extracts were also assessed for their antibacterial activity [112]. Extracts demonstrated powerful activity against Gram-positive and Gram-negative bacterial strains, such as *S. aureus*, *Enterococcus aureus*, *Bacillus subtilis*, *E. coli*, and *Pseudomonas aeruginosa*, while butanol and ethyl acetate extracts showed a moderate activity. The antimicrobial activity of *Cyperus* sp. extracts is mainly contributed by the essential oil components. The essential oil components from *Cyperus* spp. easily penetrate inside and create pores in bacterial cells which results in leakage of intracellular components resulting to cell death.

The antimycobacterial activity of *C. rotundus* extracts, evaluated on multidrug-resistant strains of *Mycobacterium tuberculosis*, also revealed to be prominent [113]. In addition, such extracts were used to assess their antibacterial activity and the mode of action against ampicillin-resistant *S. aureus*. The extract revealed a synergistic activity when in combination with ampicillin at the lowest inhibitory concentration. Using electron microscopy, it was observed that the combined treatment induced damages to the peptidoglycans and cell membrane, generating an increase in membrane permeability and revealing an inhibitory activity against *β*-lactamase [114]. Another study found that the fermented extracts of *C. rotundus* inhibited the growth of *P. aeruginosa*, *B. subtilis*, and *E. coli* [115]. For such reasons, these extracts may be conceived as a natural remedy against infections caused by pathogenic bacteria. More recently, copper oxide nanostructures synthesized using *C. rotundus* extracts revealed an excellent antibacterial activity against *Klebsiella pneumoniae* strains, and the observed inhibitory effects seemed to be associated with factors such as mechanical damage, oxidative damage, and genetic toxicity [116].

### 3.4. Anticancer Activity

The anticancer activity of *C. rotundus* extracts has also been assessed; the mechanism of action also elucidated the influence on genetic expression. For example, human cervical cancer (HeLa) cell lines exposed to different doses of *C. rotundus* extracts revealed morphological modifications and changes in the degree of chromatin condensation. Microarray analysis also showed that the extract led to the upregulation of 449 genes and downregulation of 484 genes, classified into different interaction pathways, with gene expression induction being associated with apoptosis and cell cycle arrest [117]. The main mechanism of anticancer activity of plant extracts is by inhibiting the cell proliferation or by inducing apoptosis in the cancerous cells. Both of these mechanisms are impaired in the cancerous cells. Plant extracts either halt the cell division or induce apoptosis of cancerous cells by activating the apoptotic factors. *C. rotundus* ethanol extracts were used to evaluate its effects on triple-negative breast cancer cells (TNBC) (negative for estrogen, progesterone receptors, and human epidermal growth factor receptor 2 (HER2) protein overexpression). As main findings, the authors stated that the chemical components present in the extracts inhibited the TNBC cell proliferation, which might be related to cell cycle arrest at the G_0_/G_1_ phase, thus inducing apoptosis by promoting Bcl-2-associated X protein (Bax) expression and inhibiting B cell lymphoma (Bcl) expression. The n-hexane extract from *C. rotundus* rhizomes also exhibited an anticancer activity on Michigan Cancer Foundation-7 (MCF-7) breast cancer cell lines, by inducing apoptosis and halting them in G_0_-G_1_ stages of the cell cycle [118].

**Figure 3:**
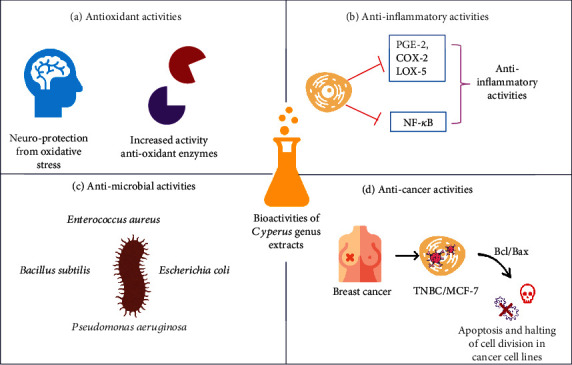
Biological activities of the extracts from *Cyperus* spp.

The cytotoxic effects of benzoquinones isolated from *Cyperus* sp. roots and tubers were also studied in adenocarcinoma gastric (AGS) and human gastric cancer cell lines. As main achievements, the authors stated that benzoquinones exerted their toxic effect by activating stress in the endoplasmic reticulum, increasing expression of C/EBP homologous protein (CHOP) (mRNA and protein levels), intracellular ROS, changes in calcium dynamics, and caspase-4 activation. The proteasome inhibition caused by hydroxyl cyperaquinone (causing cell death) was first described by the inositol-requiring enzyme 1*α*- (IRE1*α*-) independent/(PKR-like ER kinase) PERK-dependent pathway in stomach cancer cells [119]. Recently, the anticancer activity was also tested using silver nanoparticles in combination with *C. conglomeratus* extracts. The cytotoxic effect was assessed in MCF-7 breast cancer cells and normal fibroblasts using 3-(4,5-dimethylthiazol-2-yl)-2,5-diphenyl tetrazolium bromide (MTT), and a selective cytotoxicity against MCF-7 was stated, while in fibroblasts, no toxic effect was reported. Furthermore, the apoptotic effects were confirmed using annexin V-fluorescein isothiocyanate-propidium iodide (FITC-PI) and real-time PCR for apoptotic genes [120]. Various biological activities of the extracts from *Cyperus* spp. are shown in Figure 3.

### 3.5. Other Biological Activities

Six sesquiterpenes isolated from *C. rotundus* rhizome methanol extracts were analyzed as an alternative to hormone replacement therapy (HRT). Its estrogenic activity was evaluated in MCF-7 cell lines through its competitive binding to estrogen receptor- (ER-) *α* and ER-*β*. These isolated compounds revealed to be useful as an alternative to HTR [121]. Tongbi-san (TBS), the name attributed to a set of 3 herbs: *C. rotundus*, *Citrus unshiu*, and *Poria cocos*, has been used in traditional Korean medicine for dysuria. In a study, a significant reduction in body weight and a decrease in weight of epididymal and visceral white adipose tissue were found following oral administration of TBS to male mice (C57BL/6N) for 11 weeks. TBS enhanced the expression of AMP-activated protein kinase (AMPK) and inhibited the expression of transcription factors, such as CCAAT-enhancer-binding proteins (C/EBPs), sterol regulatory element-binding transcription factor 1 (SREBP1), and peroxisome proliferator-activated receptors (PPAR*γ*) in the liver and white adipose tissue of the epididymis [122].

*Cyperus eragrostis* seed extracts have also demonstrated vasodilatory properties and ability to inhibit the mammalian arginase enzyme in both an ex vivo experiment on rat aortic rings and an *in vitro* assay with purified bovine liver arginase [123]. Through an *in vivo* study in rats, mice, and chicks and *in vitro* study using isolated tissues of the jejunum and ileum of rabbits and rats, the antispasmodic, antidiarrheal, and antiemetic effects of *Cyperus niveus* Retz. were assessed. The presence of flavonoids, phenols, alkaloids, tannins, saponins, and glycosides in the extracts was described to be responsible for the significant inhibition of diarrhea in rats and for the marked decrease in the intestinal motility of mice [124].

*Cyperus articulatus* ethanolic extracts revealed to prevent pentylenetetrazol-induced seizures (PTZ) and to increase the gamma aminobutyric acid (GABA) levels in mice with PTZ [125]. Other preclinical (*in vivo* and *in vitro*) were experiments performed with *C. rotundus* to evaluate its effect on gastrointestinal, bronchial, and vascular disorders, as well as pain, emesis, pyrexia, and bacterial infections. A study revealed that the crude extract from *C. rotundus* has remarkable spasmolytic, bronchodilator, and vasodilatory effects, possibly through blockade of calcium channels [126]. A recent study evaluated the antiulcer potential of methanol and ethyl acetate extracts of *Cyperus alternifolius* L. rhizomes and aerial structures in fasted rats with orally administered indomethacin (30 mg/kg). The extracts led to a significant reduction in the number of ulcers and TNF-*α* content in the stomach. Histopathological examination revealed an improvement in damaged mucosa, with the effect generated by tubers being more effective than that of the control ranitidine [127].

## 4. Health-Promoting Effects: Data from Clinical Findings

As previously referred (Section 1), *Cyperus* spp. have been used over the years in folk medicine around the world to prevent and even treat different medical affections. *C. rotundus* is the most widely used and exploited species from this genus, and the metabolites present in this species are currently well elucidated [128, 129], so as their bioactive effects, analyzed through a number of investigations [130]. For example, the combined effects of *C. rotundus* with other medicinal plants taken as capsules of dehydrated plants or in the form of decoction were found effective against overweight, obesity, Alzheimer's disease (AD), depression, and rheumatoid arthritis. However, no clinical trials on *C. rotundus* used alone could be found.

An Ayurvedic polyherbal formulation, called Trimad, composed of *C. rotundus* tubers, *Embelia ribes* Burm.f. fruits and *Plumbago zeylanica* L. roots traditionally used for the management of overweight and obesity, was investigated by Salunke et al. [131] in 20 overweight and obese individuals. In this clinical study, the authors aimed to assess the effect of the aqueous extract of triphala (formulation made from *Emblica officinalis* L., *Terminalia bellirica* (Gaertn.) Roxb., and *Terminalia chebula* Retz.) (two tablets of 500 mg, twice a day after meals), Trimad, and placebo (dextrin) for the management of obesity and overweight, over 90 days. Significant differences were stated in visceral and subcutaneous fat, as well as other benefits, like positive bowel regulation and a decrease in fatigue in patients who received triphala [131]. Similarly, an Ayurvedic polyherbal mixture (no common name was mentioned) proved to be effective in the management of type 2 diabetes, with findings being compared to metformin effects in a 6-month treatment. The capsules, consisting of *Berberis aristata*, *C. rotundus*, *Cedrus deodara*, *Emblica officinalis*, *Terminalia chebula*, and *Terminalia bellirica*, following HPLC analysis revealed the presence of berberine (1.27%), quercetin (0.01%), and gallic acid (3.09%). The randomized study included 93 participants, and 48 of them received 3 g of the herbal treatment for 6 months. The study concluded that the formulation had a positive effect on blood glucose levels, on glycosylated hemoglobin, and on the lipid profile of the patients without showing any adverse effects [132]. Finally, another traditional Ayurvedic formulation with *C. rotundus* [133] was found to be useful for rheumatoid arthritis. The herbomineral formulation (5 g of each plant), consisting of *Vitex negundo*, *C. rotundus*, *Nyctanthes arbor-tristis*, *Smilax glabra*, *Delphinium denudatum*, and *Withania somnifera*, was combined with Maha yogaraj Guggulu Vaiswanar churna and Simhanada Guggulu. No information on its safety or chemical composition was provided in the article, despite the fact that patients (39%) who receive the preparation twice a day before meals for 1 year exhibited a good response to the treatment.

The possible use of *C. rotundus* against neurocognitive disorders has also been reported. The combination of *C. rotundus* with *Crocus sativus* and *Astragalus membranaceus* honey was assessed for its ability to treat a major neurocognitive disorder. A double-blind clinical trial on 60 patients previously diagnosed was performed. The intervention group took two daily capsules of 500 mg each one of the combinations for three months. The results indicated that the combination could be useful to improve the cognitive and depression score. However, the extract used was not characterized, and the preparation method was not indicated [134]. The traditional Iranian herbal medicine, Davaie Loban, composed of *C. rotundus*, *Zingiber officinale*, *Acorus calamus*, *Piper nigrum*, and *Boswellia carterii* has also been tested against AD. In the study, 24 patients with mild to moderate AD took 500 mg of the capsules 3 times daily for 12 weeks. Results showed that the herbal mixture might have an improvement in the memory of patients. However, despite the safety of the administered mixture which was tested, the preparation method was not detailed [135]. In traditional Chinese medicine, the Guilpi decoction, an herbal mixture, is used given its ability to regulate blood pressure and as an antidepressant [136]. So, Zhuang et al. [137] tested the effect of Guilpi decoction in 120 elderly patients affected with hypertension and depression. The decoction was prepared as follows: 15 g of a mixture formed by *Codonopsis pilosula*, *Atractylodes*, *Angelica sinensis*, membranous milkvetch root, *Polygala tenuifolia*, and *Arillus longan*; 20 g of *Poria cocos* and *magnesium*; 30 g of calcined Os Draconis, calcined oyster shell, light wheat, and concha haliotidis; and 10 g of elecampane, fresh ginger, Chinese date, colla corii asini, mint, *Albizia julibrissin*, *C. rotundus*, jasmine, and *Coptis chinensis* as well as spina date seed of 40 g. The mixture was decocted, and patients received 400 ml of extract for a month. The control group was treated with sertraline. Results concluded that the mixture exerted a curative effect and improved the patients' quality of life by alleviating depression symptoms, although no significant differences were reported in blood pressure.

Some other clinical trials have been performed using the extracts of *Cyperus* spp., but the species investigated were not specified in a large amount of cases. These are the cases of mixtures made with *Cyperus* for sexual dysfunction [138], as anti-inflammatory and analgesic [139] agents and even as lactation inducers [140]. Another critical limitation present in many clinical trials is that in most cases, as previously listed, the herbal mixture was not characterized, and when a chemical characterization was reported, only some polyphenolic compounds were analyzed. In a case, the herbal mixture was consumed as an aqueous extract, but the polyphenol analysis by HPLC was done in a methanol/water extract. Therefore, the active metabolites responsible for the therapeutic action remain unknown, and the possible synergistic and antagonistic interactions among the plant constituents were not addressed neither described. In addition and also worth noting is that few researches have done a microbiological or safety study on the dehydrated plants when consumed in the form of capsules of dried plants. However, the presence of pathogens, spores, heavy metals, and even aflatoxins was not described in the preparations, which otherwise would have been a potential risk for patients. Moreover, such clinical trials were done with a few patients, and in most of them, both treatment monitoring and fidelity were not precise. Therefore, there is an urgent need to design more robust and reproducible clinical trials to prove the potential health benefits of *Cyperus* spp., which have been widely described via a plethora of *in vitro* and *in vivo* tests.

## 5. Safety and Adverse Effects

Due to its richness in chemical constituents, *Cyperus* plants have been widely used in folk medicine for multiple affections [100, 141, 142]. Thorough screening of literature available on *Cyperus* plants as a popular remedy among various ethnic groups, researchers have increasingly explored their therapeutic potential [143]. However, it is also of extreme interest to evaluate the toxicological aspects of botanical drugs and products for their reliable and safe usage among consumers.

Toxicological data from *C. rotundus* extracts have been reported by several investigators [144]. Most of them reported the use of *Cyperus* extracts as safe [100, 109], with no side effects or even only minor side effects being reported. For example, *C. rotundus* extracts were studied *in vivo* for toxicity and biochemical activities in mice and rats. The lethal dose LD_50_ of *C. rotundus* root extract, when administered intraperitoneally, was 90 g/kg [145, 146]. Ethanolic extract of dried roots of this plant administered to mice of both sexes showed LD_50_ > 0.5 mg/kg [147]. Aqua-ethanolic (1 : 1) extracts of rhizome administered to mice of both sexes produced LD_50_ of 681.0 mg/kg [148]. In turn, the LD_50_ of *Cyperus* essential oils was 5000 mg/kg in rats [149]. Other studies reported that a single oral administration of *C. rotundus* ethanolic extract at 5000 mg/kg did not produce signs of toxicity, behavioral changes, mortality, or differences on gross appearance of internal organs in the animals. In subacute toxicity, all rats received a repeated oral dose of 1000 mg/kg of the ethanolic extract for 14 days. The parallel group received the ethanolic extract in the same period but was kept for further 14 days. Application did not produce any effects or reversibility of toxic effects. Thus, it was concluded that the extract did not cause changes in terms of general behaviors, mortality, weight gain, and hematological and clinical blood chemistry parameters in comparison to the control group [150]. Another research also confirmed the safety of *Cyperus* extracts. *C. rotundus* methanolic extracts in mice at doses of 250 and 500 mg/kg body weight showed no toxic effects [151]. On the other side, Lemaure and coworkers [152] documented that the administration of 45 or 220 mg/kg/day of *C. rotundus* tubers' hexane extracts for 60 days in rats stimulated a significant reduction in weight gain but without toxic effects. Raut and Gaikwad [153] also observed no toxicity symptoms following the administration of *C. rotundus* crude extract at different concentrations and oral doses. Jebasingh et al. [154] performed acute toxicological studies with *C. rotundus* extract and found no mortality or morbidity up to 2000 mg/kg body weight in rats. Toxicity studies also revealed no changes in food, water consumption, and body weight of animals with an increase in white blood cell count and hemoglobin level as well as improvement in kidney and liver function. Krisanapun et al. [155] carried out the acute toxicity test of *C. rotundus* water extracts in rats and reported the single oral LD_50_ > 5 g/kg body weight. The *C. rotundus* extract used at three doses, 10, 100, and 1000 mg/kg, did not exhibit any sign of toxicity. However, it was observed that at 1000 mg/kg, motor activity was slightly decreased. The effects of *C. rotundus* extract were also assessed on different biochemical parameters (glucose, lipid profile, cardiac enzymes, liver enzymes, and kidney function test). Liver enzymes were found normal, and a nonsignificant increase in serum bilirubin, gamma-glutamyl transferase (GGT), and serum glutamic-pyruvic transaminase (SGPT) was recorded. Also, hematological studies did not show any significant toxic changes, and histopathological examination confirmed that the tested extract was safe and nontoxic [156]. Finally and also worth noting is the use of *Cyperus* spp. for biotechnological purposes, namely, regarding its use as a functional food additive. For example, Carvalho Barros and coworkers [157] evaluated the replacement of beef fat in beef burgers using a tigernut (*C. esculentus*) oil emulsion, to reduce total fat and saturated fatty acids in the studied samples. As main findings, the authors stated that total replacement of beef fat using tigernut oil emulsions in beef burger resulted in a well-accepted and healthier meat product with reduced total and saturated fat contents, as well as increased unsaturated fatty acids [157]. However, further studies are needed to further explore other *Cyperus* sp. agroindustrial and biotechnological potentialities.

## 6. Conclusions and Perspectives

Across the diverse traditional systems of medicine, *Cyperus* sp. is popularly employed as a potent ethnomedicine owing to its plethora of pharmacological attributes, *viz.*, antioxidant, anti-inflammatory, neuroprotective, antidepressive, antiarthritis, antiobesity, antimicrobial, anticancer, vasodilator, spasmolytic, bronchodilator, and estrogenic properties. This wide array of biological activities is closely linked to the presence of phytochemicals such as *α*-cyperone, *α*-corymbolol, *α*-pinene, caryophyllene oxide, cyperotundone, germacrene D, mustakone, and zierone. However, its wide-ranging use in folk medicine and expansive pharmacological properties are not corroborated with incontrovertible evidences employing animal models, where despite the bioactive phytochemicals of *Cyperus* spp., they have been well-deciphered. Comprehensive investigations on the pharmacological efficacies of isolated compounds are still inadequate. Moreover, structure-activity analyses on the obtained phytoconstituents have also uncovered the perception of the underlying molecular insights of action of its active extracts and/or phytochemicals. On the other side and although toxicological data have indicated the use of *Cyperus* spp. as safe and effective, conclusive studies encompassing its clinical, toxicological, and safety features are still sparse. In addition and given that reported clinical trials lack herbal mixtures' characterization containing *Cyperus* spp., further studies are needed to explore the precise active constituents. Moreover, the subchronic toxicity as well as their interactions with commonly used conventional drugs to assure a safe and long-term consumption by human subjects also needs further attention of the researchers. Lastly and not least interesting to underline is that, considering the extensive pharmacological potential of *Cyperus* spp., more in-depth research is needed to attain a greater clarity of its mechanism of action.

## Figures and Tables

**Table 1:** Examples of folk medicinal uses of a selection of *Cyperus* species.

Plant species	Country/region	Plant part (s)	Traditional use	Instruction	Reference
*Cyperus rotundus* L.	North-West Himalaya/India	Roots	Skin diseases	Decoction prepared by burning and adding the ash of fresh leaves of *A. baccifera* (10 g) and *C. rotundus* roots (10 g) and fresh ginger (5 g) in sesame oil.	[7]
	Pakistan/India	Tubers	Diabetes	10–12 g of dry tuber powder administered daily twice for 2–3 months.	[17]
	India	Whole plant	Menstruation problem	Juice of the *Citrus maxima* fruit (100 ml) and 30 g dried powder of *C. rotundus* is taken once daily for a week.	[18]
	Tamil Nadu/India	Tubers	Snake bite	Paste of leaf and root bark of *Albizia amara*, root bark of *Jasminum angustifolium*, and tubers of *C. rotundus* is heated with oil and applied externally on affected places for 10 days.	[19]
	India	Roots/tubers	Urinary trouble-stone removal	Decoction of the plant is used.	[20, 21]
	India	Whole plant	Epilepsy	Plant decoction (10 ml) with 5 ml of honey is orally administered to treat epilepsy.	[22]
	India	Roots	Cholera	Roots are boiled with equal quantity of mint and given for cholera.	[23]
	India	Roots	Pimples	Roots along with turmeric and curd are made into a paste which is applied on the face for pimples and beautification	[23]
	India	Roots	Increase lactation	Paste of the roots is applied on breasts to increase lactation.	[23]
	North-West Himalaya	Roots	Intermittent fevers	The decoction prepared from 10 g of *C. rotundus* roots and 5 g of fresh ginger is used.	[24] Das, & Misra [25]
	India	Tubers	Dermatitis	The decoction prepared from tuberous roots of *C. rotundus* and leaf of *Trichosanthes anguina* is taken orally to cure dermatitis.	[25]
	India	Tubers	Dysentery	The tuberous root of *C. rotundus* with other plants which are orally used to treat dysentery is taken in three doses to cure dysentery.	[25]
	India	Tubers	Indigestion disorders, stomachache	A powder was prepared from 10 g of tuber of *C. rotundus*, 10 g stem bark of *Holarrhena antidysenterica*, and 10 g of *Zingiber officinalis* after being sun dried. 30 g powder is given internally along with 250 ml of buttermilk twice daily till cure.	[26]
	India	Tubers	Vaginal discharge	Tubers crushed with *Abutilon indicum* leaves and sufficient quantity of *Cuminum cyminum* seeds; extract administered daily twice for three days.	[27] Jahan et al. [28]
	India	Whole plant	Loss of libido in men	Leaves of *Psidium guajava*, leaves of *Punica granatum*, and whole plants of *C. rotundus* are mixed, warmed, and macerated to obtain juice. 1/2 cup of the juice is taken with 10–15 drops of honey twice daily for 3 days.	[28]
	India	Tubers	Constipation	1/2 cup of juice obtained from macerated tubers is taken three times daily.	[28]
	India	Whole plant	Bone fracture	Whole plant of *C. rotundus* and 7 slices of ginger are crushed and made into a paste. The paste is warmed and applied to fractures.	[29]
	India	Tubers	Jaundice	Fresh rhizome with tuberous root of C*. rotundus* and fruits of *Phyllanthus emblica* are taken in equal quantities and ground. 2 spoonfuls of paste mixed in a glass of water are administered daily once for 8 days.	[30]
	India	Bark	Malaria	The decoction is prepared from a mixture of 200 g of rhizome of *Costus speciosus*, 200 g bark of *C. rotundus*, and 200 g bark of *Azadirachta indica*. 2–4 spoons of decoction were prescribed after meal for 15 days.	[31]
	India	Tubers	Bronchitis	Tubers of *C. rotundus* and leaves of *Tinospora cordifolia* with fruits of *Pergularia daemia* are ground. 2 spoons of paste with honey are orally administered twice daily for 30 days.	[32]
	China	—	Coughs	—	[33]
	Rarotonga	Tubers	Sore throat	Twenty to thirty tubers of *C. rotundus* and a handful of *Pandanus tectorius* bark which is crushed into the water of four green coconuts. Half the mixture is drunk hot, and the remainder cold. The treatment lasts for three days.	[33]

*Cyperus javanicus* Houtt.	Rarotonga	Leaves	Fractures/sprains	Leaves without flowers are pounded and squeezed into a small basin of water. The treatment lasts for three days.	[33]
	Rarotonga	Leaves	Irregular menstrual	Leaves with those of several other herbs.	[33]

*Cyperus brevifolius* (Rottb.) Hassk.	Malaysia	Tubers	Sore legs	—	[33]

*Cyperus kyllingia* Endl.	Rarotonga	Tubers	Oral thrush	Tubers of *C. kyllingia*, 4 *Aleurites moluccana* inside nuts, and a handful of the aerial roots of *Ficus prolixa* are pounded then squeezed through a cloth into a liter of water.	[33]

*Cyperus monocephalus* Roxb.	Philippines	Tubers	Dermatosis	Decoction is prepared from tuberous root.	[33]
	Tami Islands	Tubers	Ringworm	Decoction of tubers prepared by adding lime.	[33]

*Cyperus compressus* L.	India	Roots	Helminthiasis	Powdered roots orally administered.	[16]

*Cyperus articulatus* L.	Central Africa Republic	Tubers	Headache, migraine	Decoction is prepared from tuberous root.	[34]

*Cyperus pedunculatus ***(**R.Br.) J.Kern	West Africa	Stem and leaves	Diarrhea, kidney disease, fever, pain, and inflammations	Extract is made from the whole plant	[35]

*Cyperus nitidus* Lam.	South Africa	Rhizomes	Respiratory and digestive disorders	Extract is made from the rhizomes	[36]

*Cyperus sexangularis* Nees	South Africa	—	Asthma, fatigue, fever, pneumonia, and TB	—	[37]

*Cyperus sexangularis* Nees	South Africa	Roots	Antimicrobial, emollient, diuretic, stimulant, anthelmintic, and analgesic treatment	Extract is made from the roots	[38]

*Cyperus kilimandscharicus* Kük.	East Africa	Roots	Various animal diseases	Extract is made from the roots	[39]

*Cyperus latifolius* Poir.	East Africa	Roots	Tuberculosis and related ailments	Extract is made from the roots	[40]

*Cyperus maculatus* Boeck.	West Africa	Tubers	Cattle worms	—	[41]

*Cyperus natalensis Hochst.*	South Africa	Roots	Treatment of gynaecology and obstetric complaints	Decoction is prepared from the roots	[42]

*Cyperus erectus* (Schumach.) Mattf. & Kük.	South Africa	—	Reduces foot swelling	Ground plant is used for the medicinal purposes	[43]

*Cyperus mundii* (Nees) Kunth	Madagascar	—	Treatment of evacuation of the placenta, tuberculosis, and paludism	Whole plant extract	[44]

*Cyperus esculentus* L.	Oaxaca, Santa María Tecomavaca	Roots	Depression	Root extracts	[45]

*Cyperus flavescens* L.	Oaxaca, Santa María Tecomavaca	Roots	Depression	Root extracts	[45]

**Table 2:** Phytochemicals present in different *Cyperus* species.

*Cyperus* species	Chemical constituents	Plant part	References
*Cyperus articulatus* L.	*α*-Campholenal, *α*-corymbolol, *α*-cyperone, *α*-pinene, cyperol, cyclocolorenone, *β*-copaen-4-*α*-ol, p-cymene, caryophyllene oxide, corybolane, cyperotundone, limonene, thuja-2,4(10)-diene, *trans*-pinocarveol, p-mentha-1,5-dien-8-ol, myrtenal, mustakone	Thick rhizomes	[49, 50]
*Cyperus conglomeratus* Rottb.	Saponins, steroids, tannins, triterpenes	Whole plant powder	[51]
*Cyperus distans* L.f.	Artemisia ketone, *α*-cyperone, cyperene, *α*-pinene, 1,8-cineole, caryophyllene oxide, endesma-2,4,11-triene, humulene epoxide II, germacrene D, pinocarveol, myrtenol, nor-copernone, zierone	Rhizomes	Lawal et al. [52]
*Cyperus esculentus* L.	*β*-Pinene, cymene, cyperene, coumaran, cyperotundone, p-vinylguaiacol, vanillin, cyprotundone	Rhizomes	Gugsa & Yaya [46]
*Cyperus longus* L.	*α*-Caryophyllene oxide, *β*-himachalene, *β*-caryophyllene oxide, aristolone, humulene oxide, irisone, longiverbenone, viridiflorol	Whole plant powder	Memariani et al. [53]
*Cyperus rotundus* L.	Isobutyl lactate, thiazol-4(5H)-one.5-(4-nitrobenzylidenol)-2-phenyl, *cis*-pinen-3-ol, *trans*-p-mentha-2,8-dienol, pyranone, *cis*-10-nonadecenoic acid, *β*-santalol, *α*-copaen-11-ol, *β*-vatirenene, elema-1,3-dien6a-ol, *β*-nootkatol, *cis*-13,16-docasadienoic acid, 25,26-dihydroxy-vitamin D3	—	El-Wakil et al. [54]
